# Co-occurrence networks reveal the central role of temperature in structuring the plankton community of the Thau Lagoon

**DOI:** 10.1038/s41598-021-97173-y

**Published:** 2021-09-03

**Authors:** Thomas Trombetta, Francesca Vidussi, Cécile Roques, Sébastien Mas, Marco Scotti, Behzad Mostajir

**Affiliations:** 1 MARBEC (Marine Biodiversity, Exploitation and Conservation), Univ Montpellier, CNRS, Ifremer, IRD, Montpellier, France; 2 MEDIMEER (Mediterranean Platform for Marine Ecosystems Experimental Research), OSU OREME, Univ Montpellier, CNRS, IRD, IRSTEA, Sète, France; 3 GEOMAR Helmholtz Centre for Ocean Research Kiel, Kiel, Germany

**Keywords:** Ecology, Environmental sciences, Hydrology, Ocean sciences

## Abstract

To identify the environmental factors that drive plankton community composition and structure in coastal waters, a shallow northwestern Mediterranean lagoon was monitored from winter to spring in two contrasting years. The campaign was based on high-frequency recordings of hydrological and meteorological parameters and weekly samplings of nutrients and the plankton community. The collected data allowed the construction of correlation networks, which revealed that water temperature was the most important factor governing community composition, structure and succession at different trophic levels, suggesting its ubiquitous food web control. Temperature favoured phytoplanktonic flagellates (Cryptophyceae, Chrysophyceae, and Chlorophyceae) and ciliates during winter and early spring. In contrast, it favoured Bacillariophyceae, dinoflagellates, phytoplankton < 6 µm and aloricate Choreotrichida during spring. The secondary factors were light, which influenced phytoplankton, and wind, which may regulate turbidity and the nutrient supply from land or sediment, thus affecting benthic species such as *Nitzschia* sp. and *Uronema* sp. or salinity-tolerant species such as *Prorocentrum* sp. The central role of temperature in structuring the co-occurrence network suggests that future global warming could deeply modify plankton communities in shallow coastal zones, affecting whole-food web functioning.

## Introduction

Environmental forcing factors play a central role in driving plankton community composition and dynamics in marine and freshwater ecosystems. At a global scale, along latitudinal gradients, species distribution and community composition depend on abiotic conditions, such as temperature, light, and nutrients^1^. On the other hand, at the local level, food web structure is more affected by biotic processes such as predation, competition, population growth and behaviour^2^, which are constrained by environmental conditions. However, in highly dynamic systems subject to intense environmental stressors, physico-chemical forcing factors may play a predominant role in shaping communities^3,4^. Furthermore, the plankton community’s response to environmental forcing factors in these systems is challenging to determine, as these factors can be influenced by various elements and are often linked together^5^. For example, alongshore wind in coastal ecosystems triggers deep, cool and nutrient-rich water upwelling, thus influencing plankton communities^6,7^. Consequently, the conjunction of wind direction and speed, column mixing, water temperature and nutrient concentration changes explains the plankton response during these events. Therefore, it is essential to study multiple environmental parameters together in particularly highly dynamic systems, as they can be tightly linked together.

Shallow coastal waters, including coastal lagoons, estuaries, seagrass beds, and coral reefs, are highly dynamic and often exposed to extreme environmental events. Community composition and structure in these zones are driven by environmental forcing factors, mainly because the zones occupy the interface between land and sea^8^. These factors influence plankton communities directly or indirectly. For instance, river runoff transports nutrients and terrigenous organic matter, influences water turbidity, and modulates phytoplankton and bacterial production^9,10^. Seawater currents or tides recirculate nutrients, which provide critical elements for the food web and enrich local communities of offshore organisms^11–13^. These water inputs and precipitation induce significant salinity variations that affect the plankton community^14,15^. Moreover, the shallow depth of these coastal waters makes them particularly sensitive to the action of wind and temperature variations. This influences the stability of the whole water column (mixing and stratification) and indirectly alters community dynamics through sediment and nutrient resuspension and turbidity increases^16–18^. Furthermore, coastal ecosystems exhibit relatively low inertia, and due to their shallow nature, environmental changes can be rapid^3,19^. However, few studies have investigated the environmental forcing factors structuring the plankton community composition and structure in shallow coastal waters, preventing a deep understanding of plankton food web functioning.

Correlation analyses are used to build networks mapping the relationships between species^20–22^. The analysis of these networks is practical for formulating hypotheses about the most critical mechanisms underlying community assembly. For example, it may suggest the prevalence of top-down forces or a shift from grazing chain-dominated food webs to systems where the microbial loop prevails^22^. In addition to being routinely applied to model planktonic food webs, network analysis is also a powerful tool for studying the effects of abiotic forcing factors on plankton food webs^23,24^. It can, for example, shed light on the role of complex physico-chemical changes responsible for shifts in planktonic food webs triggered by environmental forcing, for example, anthropogenic hydrology alterations in natural coastal lagoons^24^.

The present study’s objective was to investigate environmental factors associated with changes in plankton community composition and structure in a highly dynamic system, Thau Lagoon, a shallow coastal lagoon along the northwestern Mediterranean coast. Correlation networks were constructed using organism abundances and environmental metrics in different seasons. Environmental multiparameter monitoring and weekly samplings for planktonic abundance quantification were carried out from winter to late spring during two consecutive years, 2015 and 2016. These two years were very distinct and presented different characteristics. While 2015 was a typical year in terms of climate, with a high thermic amplitude from winter to spring, 2016 was characterised by anomalously high water temperatures during winter, with the warmest winter ever recorded in southern France (http://www.meteofrance.fr/climat-passe-et-futur/bilans-climatiques/bilan-2016/hiver#:~:text=Sur%20l'ensemble%20de%20la,aequo%20(%2B%201.8%20%C2%B0C). The present study follows two previous works that focused on the environmental forcing factors triggering phytoplankton blooms initiation^19^ and on the microbial food web interactions in Thau Lagoon^22^. These studies found that the particular climatic characteristics of 2016 led to a the dominance of small size phytoplankton in the community^19^. These conditions might also have favoured an increase in the number of biotic interactions among smaller organisms^22^, even if the links between environmental variables and plankton community composition were not explicitly investigated. Thus, the present study will, for the first time, quantitatively link multiple environmental variables to plankton community composition and structure in a characteristic shallow coastal lagoon.

## Results

### Temporal dynamics of planktonic organisms

**Figure 1:**
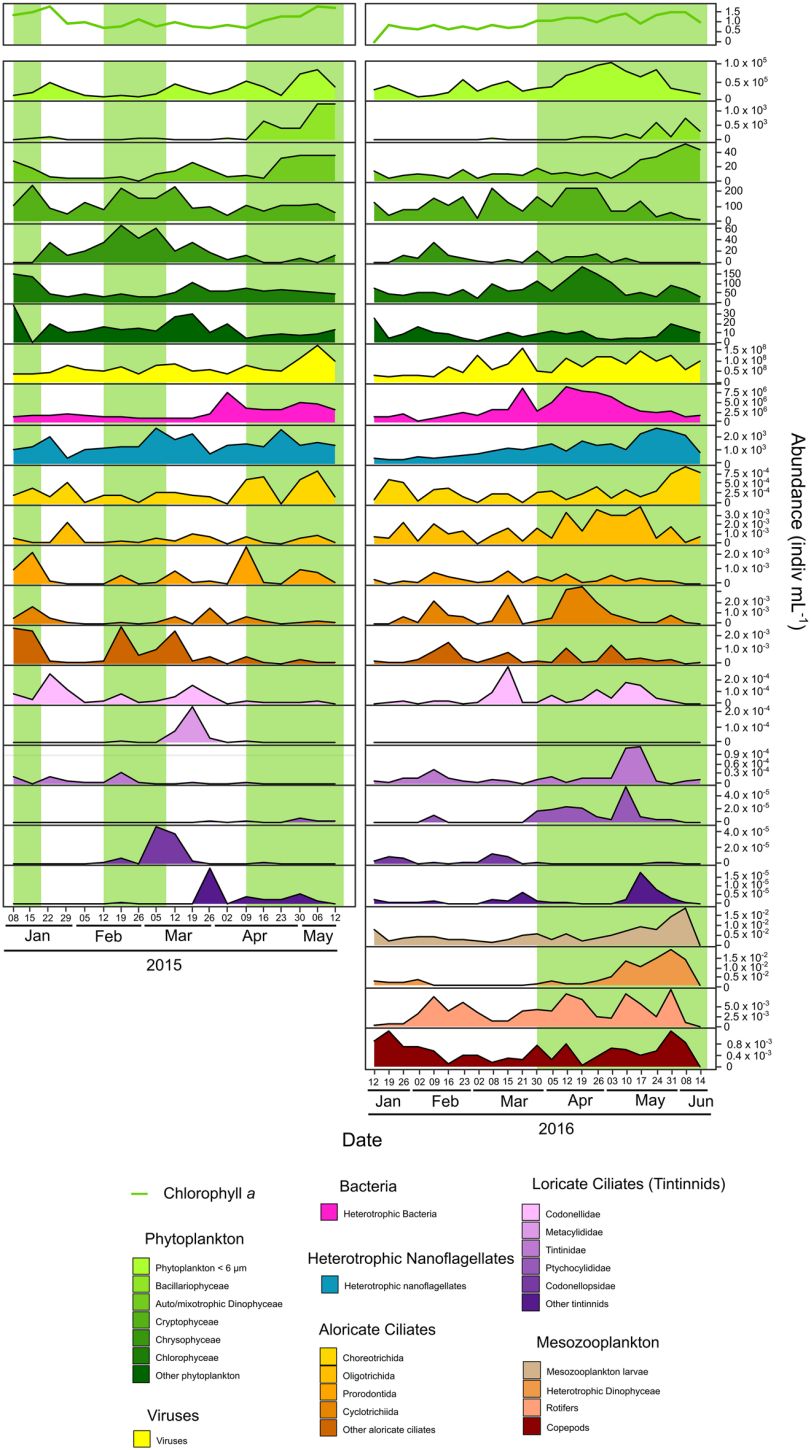
Dynamics of Chl *a* and plankton abundances in 2015 (left) and 2016 (right). The green background represents bloom periods, and the white background represents non-bloom periods.

A previous study focused on the role of environmental forcing factors in determining bloom initiation^19^. The study used daily mean Chl *a* fluorescence as a measure of phytoplankton biomass and allowed distinguishing phytoplankton bloom and non-bloom periods (Fig. 1). Such distinction was based on the number of consecutive days of net growth rates. Three bloom periods occurred in winter, early spring and late spring in 2015 (Fig. 1; for details, see Trombetta et al. 2019)^19^. For 2016, only one long spring bloom was found. The dynamics of various planktonic groups in Thau Lagoon are shown in Fig. 1. These dynamics showed that phytoplankton < 6 µm, Bacillariophyceae, and auto/mixotrophic Dinophyceae mainly peaked during spring in both years. Cryptophyceae, Chrysophyceae, Chlorophyceae, and other phytoplankton groups mainly peaked during winter and early spring in both years. The abundance of viruses and bacteria slightly increased from winter and reached its highest level from early spring to late spring in both years. The abundance of heterotrophic nanoflagellates (HFs) was always high in 2015 and peaked in winter, early spring and late spring, while their abundance was low in winter 2016 and slightly increased to reach a maximum value in late spring. Aloricate ciliates and tintinnids exhibited differences between 2015 and 2016. Choreotrichida were the only taxa peaking during late spring of both years. Oligotrichidae, Prorodontida, Cyclotrichiida, Codonellidae, Metacylididae, Tintinnidae, and Codonellopsidae peaked in winter or early spring in 2015 but had low abundance during late spring. These groups, except Prostomatea and Metacylididae, peaked from spring to mid/late spring in 2016. The abundance of mesozooplankton larvae and heterotrophic Dinophyceae, analysed only in 2016, was low from winter to early spring and peaked towards the end of spring. Correspondent equivalent size diameter (ESD) class dynamics for 2015 and 2016 can be visualised in Supplementary Figure 3. The temporal dynamics of each taxon for 2015 and 2016 can be visualised in Supplementary Figure 4 (grouped by taxonomy) and Supplementary Figure 5 (grouped by ESD).

### Plankton community composition and environmental forcing factors

**Figure 2:**
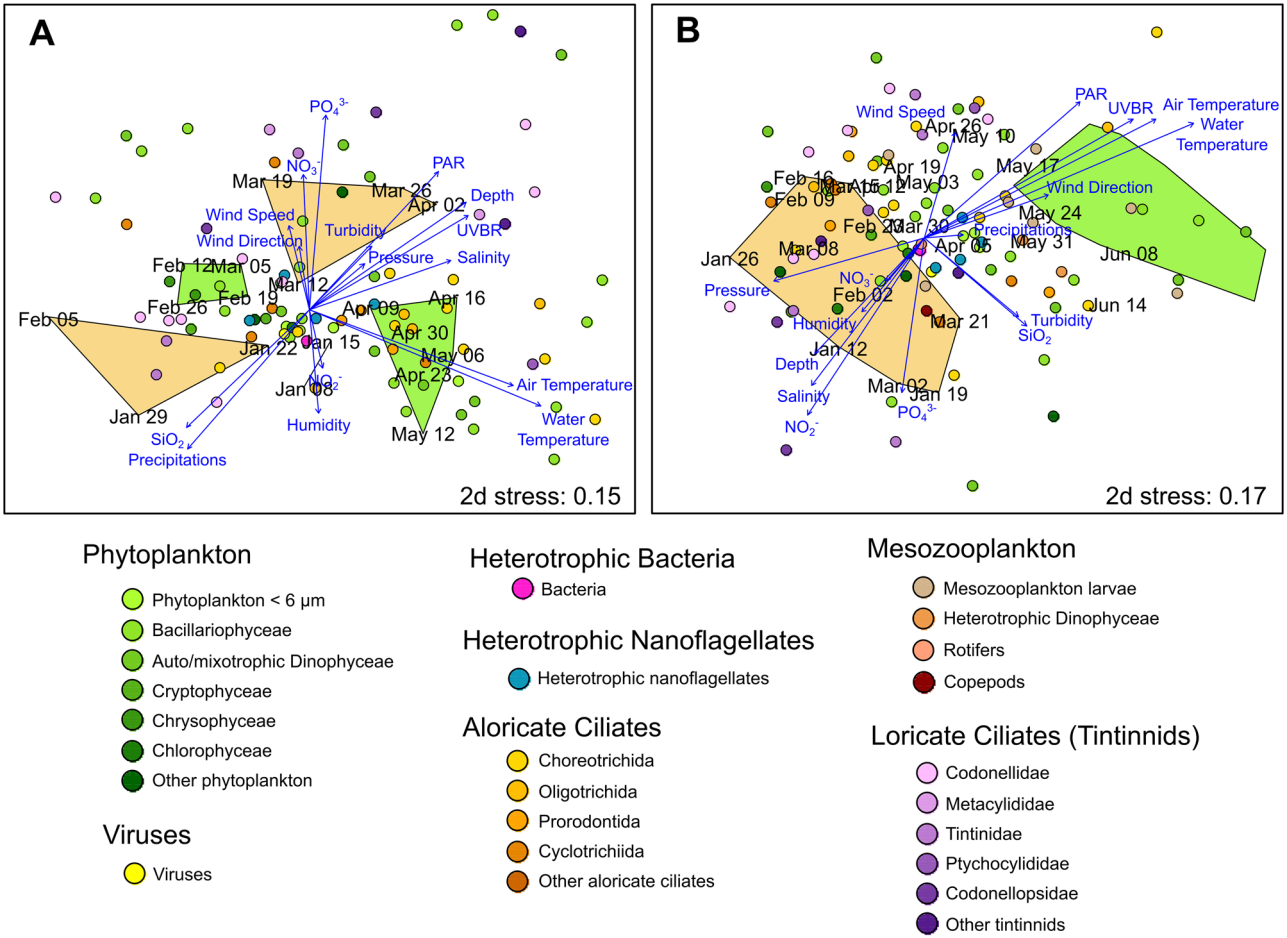
Non-metric multidimensional scaling (nMDS) of plankton community composition and environmental variables in 2015 (**A**) and 2016 (**B**). Plankton taxa are shown as circles, with colours reflecting the taxonomy depicted in the legend under the charts. Sampling dates are in black. Orange polygons delimit non-bloom periods, and green polygons delimit bloom periods. Blue arrows present the projections of environmental parameters. The proximity between environmental parameters or taxa and sampling dates indicates characteristic associations.

Non-metric multidimensional scaling (nMDS) illustrated the changes in plankton community composition along sampling dates, together with the ordination of environmental parameters (Fig. 2). A progressive transition in community composition occurred in both years from winter to spring, resulting in clear separation of the bloom and non-bloom periods. During both years, winter was characterised by low (air and water) temperatures, light (ultraviolet B radiation (UVBR) and photosynthetically active radiation (PAR)) and nutrients (NO_2_^−^, NO_3_^−^, PO_4_^3−^ and SiO_2_). Late winter and early spring (i.e., from late February to early April) displayed high wind speed and low humidity, while spring showed high light intensity and temperature. The non-bloom stages were associated with high nutrient concentrations (NO_2_^−^, NO_3_^−^, PO_4_^3−^ and SiO_2_, depending on the period), while low concentrations were found during blooms. Air and water temperature, PAR, UVBR and PO_4_^3−^ were significantly correlated with nMDS axes in both years (Table 1). Precipitation, depth and SiO_2_ were solely correlated with nMDS in 2015, and salinity and NO_2_^−^ solely in 2016.

**Table 1:** Environmental parameters on nMDS axes. Bold values indicate significant correlations between environmental parameters and nMDS axes. Scores were calculated using a linear model including both axes. Asterisks represent significance levels: * represents *p-*value < 0.05; ** represents *p-*value < 0.01; and represents *p-*value < 0.001.

Type of variable	Variable	2015		2016	
		r^2^	*p*-value	r^2^	*p*-value
Hydrological	Water temperature	**0.76**	0.0001***	**0.85**	0.0001***
	Turbidity	0.10	0.4341	0.15	0.2058
	Salinity	0.27	0.0728	**0.34**	0.0145*
	Depth	**0.44**	0.0100**	0.25	0.0561
Meteorological	Air temperature	**0.57**	0.0008***	**0.85**	0.0001***
	PAR	**0.44**	0.0109*	**0.42**	0.0036**
	UVBR	**0.42**	0.0151*	**0.57**	0.0002***
	Wind speed	0.09	0.4646	0.12	0.2849
	Wind direction	0.05	0.6552	0.17	0.1558
	Pressure	0.06	0.5883	0.25	0.0594
	Humidity	0.13	0.3326	0.09	0.3744
	Precipitation	**0.42**	0.0081**	0.01	0.8872
Nutrient	PO_4_^3−^	**0.47**	0.0054**	**0.24**	0.0441*
	NO_2_^−^	0.04	0.7061	**0.45**	0.0026**
	NO_3_^−^	0.22	0.1519	0.03	0.7130
	SiO_2_	**0.35**	0.0281*	0.18	0.1245

### Environmental and plankton community composition relationships

**Figure 3:**
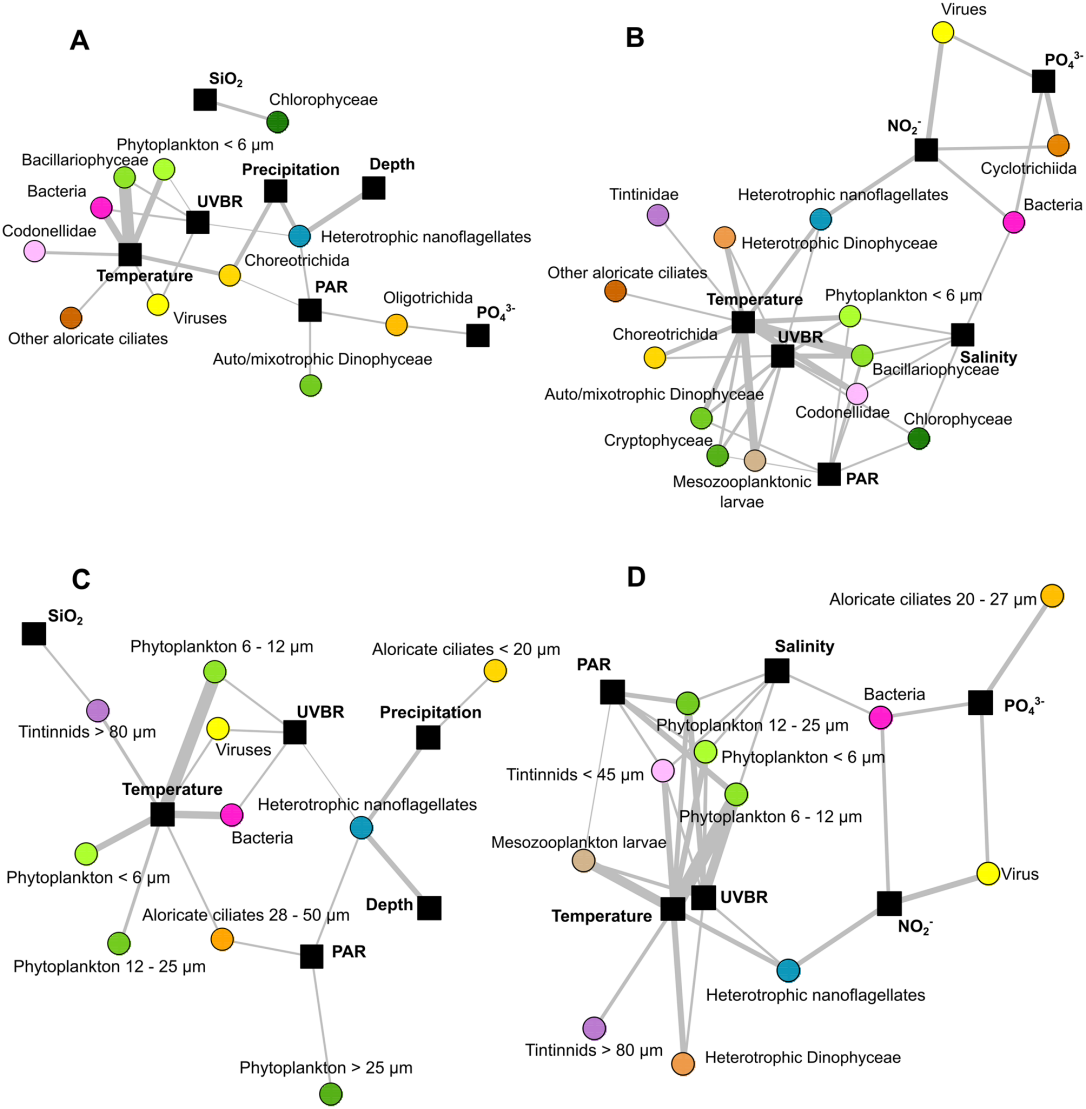
Significant correlations between environmental factors and the abundance of groups obtained considering taxonomy (**A**,**B**) and ESD classification (**C**,**D**) in the years 2015 (**A**,**C**) and 2016 (**B**,**D**). All correlations were detected by applying Mantel’s test to the most important environmental variables (i.e., those significantly correlated with nMDS axes). Node colours refer to plankton clusters. Black nodes refer to environmental parameters. The edges indicate significant correlations, and their width is proportional to the correlation strength.

Table 1 and Fig. 3 show the strongest relationships between plankton groups and the most important environmental factors. Temperature was the factor most connected to taxonomic clusters, with seven and ten significant correlations in 2015 (Fig. 3A) and 2016 (Fig. 3B), respectively. These correlations involved all types of groups, from viruses to large zooplankton. During both years, temperature was associated with phytoplankton < 6 µm, Bacillariophyceae, Choreotrichida and Codonellidae. PAR and UVBR were correlated with different taxonomic groups but mainly with phytoplankton. During both years, PAR was correlated with auto/mixotrophic Dinophyceae, while UVBR was correlated with Bacillariophyceae and phytoplankton < 6 µm. In both years, the nutrients were correlated exclusively with heterotrophic groups, and SiO_2_ was linked with Chlorophyceae in 2015 only.

Temperature was also the factor most connected to ESD clusters, with seven significant correlations in both 2015 and 2016 (Fig. 3C,D). In both years, it was associated with all phytoplankton ESD clusters (except the > 25 µm cluster in 2016). Temperature was also associated with large tintinnids (> 80 µm) and aloricate ciliates (28–50 µm) in 2015 and with tintinnids (< 45 µm and > 80 µm) in 2016. PAR and UVBR were also correlated with a large diversity of ESD clusters depending on the year.

### Correlation networks of environmental factors and planktonic organisms

**Figure 4:**
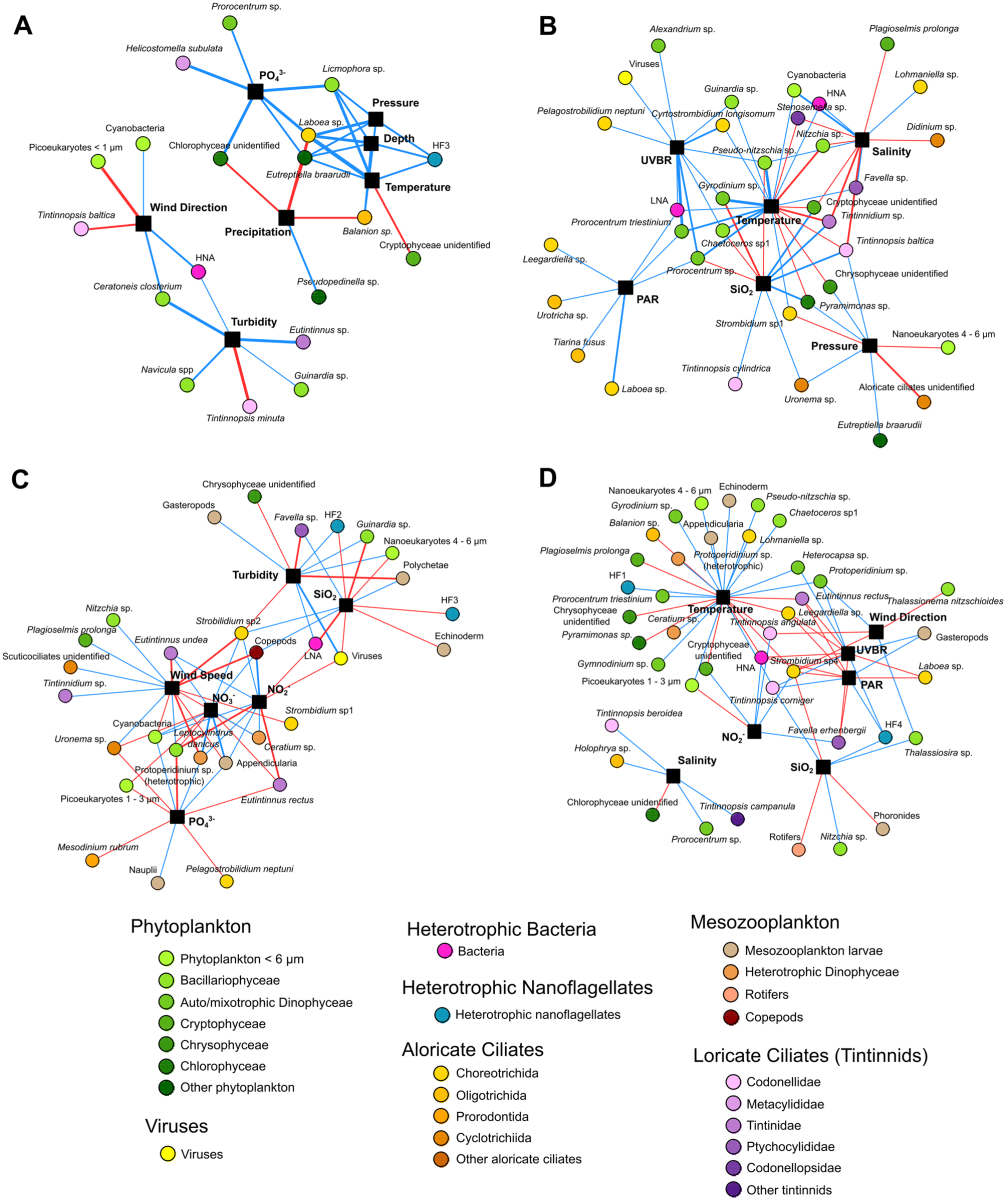
Networks of the environmental factors most correlated with the abundance of taxonomic groups in 2015 [non-bloom (**A**) and bloom (**B**)], and in 2016 [non-bloom (**C**) and bloom (**D**)]. Correlations were determined using Spearman’s rank method. Environmental factors correspond to black nodes While plankton taxa were coloured. Following taxonomic clustering. Blue and red edges represent positive and negative correlations, respectively. Correlations among plankton taxa or within the group of environmental factors are not shown for the sake of image clarity. Air temperature was not considered because it provides redundant information with water temperature. Only the most correlated environmental factors of each period were depicted while those less relevant were omitted (see Table 2).

The number of significant correlations linking each environmental factor to planktonic taxa is summarised in Table 2. The correlation networks of the most connected environmental factors and planktonic organisms are shown in Fig. 4. Preliminary analysis revealed high matching in the correlations displayed by air and water temperature with plankton taxa. Furthermore, air and water temperature share a direct, positive correlations and showed often the same correlations with other environmental factors (Supplementary Figure 6). Consequently, air temperature was omitted in Fig. 4. The most connected environmental factors during the bloom were similar between years (Table 2). Water temperature had the highest number of edges in both the 2015 and 2016 bloom periods. Furthermore, the two light wavelengths (PAR and UVBR), salinity and SiO_2_ concentration were among the most connected environmental factors in both years during blooms. During non-bloom periods, turbidity, PO_4_^3−^ concentration and wind properties (direction in 2015 and speed in 2016) were among the most connected nodes found in both years. In the non-bloom period of 2015, pressure, depth and precipitation were among the most connected factors, while nutrient concentrations (SiO_2_, NO_2_^−^ and NO_3_^−^) displayed the highest number of connections in the 2016 non-bloom period.

**Table 2:** Number of edges between the environmental factors and the plankton taxa in the bloom and non-bloom correlation networks of 2015 and 2016. Bold values emphasise the most correlated environmental factors for each period.

Type of variable	Variable	Number of edges			
		2015		2016	
		Non-bloom	Bloom	Non-bloom	Bloom
Meteorological	Air temperature	2	18	4	20
	PAR	4	7	5	9
	UVBR	4	11	3	11
	Wind speed	1	2	**14**	0
	Wind direction	5	1	3	5
	Pressure	5	8	1	2
	Humidity	0	1	5	3
	Precipitation	5	2	1	3
Hydrological	Water temperature	5	**19**	2	**26**
	Turbidity	**6**	3	10	3
	Salinity	3	12	3	5
	Depth	5	0	2	4
Nutrient	PO_4_^3−^	**6**	3	10	1
	NO_2_^−^	3	2	11	6
	NO_3_^−^	3	0	11	4
	SiO_2_	1	10	11	7

During blooms, correlation networks of both years showed the central position of temperature (Fig. 3B,D), which was connected to both autotrophs and heterotrophs. PAR and UVBR were also highly connected but mainly with heterotrophic organisms, especially aloricate ciliates and tintinnids, in the non-bloom periods of both years. During the bloom of 2015, the SiO_2_ concentration was negatively linked to four phytoplankton taxa, including two Bacillariophyceae (*Chaetoceros* sp1. and *Pseudo-nitzschia* sp.) (Fig. 4B), while during the 2016 bloom, it showed negative relationships with metazoans (rotifers and Phoronidae larvae) and the aloricate ciliate *Strombidium* sp4. (Fig. 4D).

In the non-bloom correlation networks, wind direction (2015) and speed (2016) exhibited various relationships with diverse planktonic taxa (Fig. 3A,C). Cyanobacteria were correlated with these factors in both years. In the non-bloom of 2016, wind speed shared many neighbours with NO_3_^−^, NO_2_^−^ and PO_4_^3−^, but the sign of these connections was the opposite of that for nutrients (Fig. 4C). Turbidity was among the most correlated parameters in the non-bloom periods of both years. During the non-bloom period of 2015 (Fig. 4A), turbidity was mostly connected to phytoplankton, while in the same period of 2016 (Fig. 4C), it was mainly linked to heterotrophs. Bacteria were positively correlated with turbidity in both years (HNA in 2015 and LNA in 2016). In the non-bloom period of 2015, water temperature, pressure and depth were closely and positively connected to common taxa, including phytoplankton, heterotrophic nanoflagellates and aloricate ciliates.

Spearman’s correlation networks between environmental parameters are shown in Supplementary Figure 6.

## Discussion

Phytoplankton blooms are common from winter to spring in the Thau Lagoon and observed for decades^17,19,25–29^. In the Thau Lagoon, increasing water temperature during spring drives phytoplankton bloom initiation^19^ and inter-annual warming favours interactions among small organisms and strengthens trophic cascades^22^. However, no studies were made on the role of water temperature, and other environmental factors, on the structure and succession of the entire plankton community. The present study filled this gap by considering a large diversity of taxa, ESD and functions. The findings of the present study suggest for the first time that water temperature, from winter to spring, is the most important environmental factor driving the composition, succession (Figs. 2, 3; Table 1) and species dynamics (Fig. 4; Table 2) of the whole planktonic food web in a shallow coastal lagoon. Such control encompasses various trophic levels, from phytoplankton, viruses and bacteria to heterotrophic nanoflagellates, ciliates and mesozooplankton which are detailed below. The presence of significant correlations between water temperature and organisms belonging to various trophic levels suggests extensive impacts on the entire plankton food web. Although these correlation-based results cannot describe causal mechanisms, they clarify the pervasive impact that temperature has on the structuring of the planktonic food web in a shallow coastal zone such as the Thau Lagoon. Temperature affects organisms in several ways, by (1) acting on growth rates and metabolism, (2) regulating trophic interactions^30,31^, and (3) influencing abiotic processes in ecosystems (e.g. water column mixing and stratification)^32^.

Water temperature directly influences the metabolism of organisms. Specifically, it accelerates the metabolism and consequently the growth rates of organisms^30^. In the present study, in most of the cases, water temperature was linked to taxa by positive correlations (Fig. 4), meaning that their abundance increased when water temperature increased. For instance, the phytoplankton species *Chaetoceros* spp. (two species). and *Pseudo-nitzschia* sp. mainly appeared during spring blooms (Supplementary Figure 4) of both years and were positively correlated with water temperature (Fig. 4). In Thau Lagoon and in Mediterranean coastal waters, *Chaetoceros* spp. (two species) and *Pseudo-nitzschia* sp. are the main Bacillariophyceae components of spring phytoplankton communities, blooming between 12 and 14 °C and persisting at high abundance even when the water temperature rises above 20 °C^19,25,33^. On the other hand, the direct metabolic response is more complex than a simple rise in abundance driven by a temperature increase and might also be due to the thermal optimum of taxa and temporal thermal niches realised^34,35^. In fact, water temperature influences the composition and succession of the plankton community from winter to spring (Fig. 2; Table 1), including a large diversity of taxonomic and ESD clusters, for both heterotrophic and autotrophic groups (Fig. 3). The aloricate ciliates belonging to Choreotrichida and Codonellidae peaked on several dates from winter to spring (Fig. 1). During the 2016 bloom, the aloricate ciliates *Leegardiella* sp. and *Tintinnopsis angulata* exhibited a negative correlation with temperature, while *Lohmaniella* sp. and *Tintinnopsis corniger* displayed a positive connection with this factor. At low temperature, the abundance of *Leegardiella* sp. and *T. angulata* was high, while *Lohmaniella* sp. and *T*. *corniger* increased in abundance with increasing temperature (Supplementary Figures 2 and 4), suggesting that they have different thermal niches. Furthermore, water temperature can have a direct effect on biotic interactions^31^. There is evidence of the influence of temperature on biotic interactions of the planktonic food web, such as predation^36,37^, competition^38,39^, mutualism^40^ and parasitism^41,42^. In our case study, temperature modifications might have influenced organismal interactions and played an important role in the succession of the plankton community. The modification of grazing rates due to water temperature increases^30,43^ was pointed out several times as a major actor modifying the plankton community composition in mesocosm experiments where the temperature was manipulated^37,44^.

Water temperature also modifies the abiotic environment and has indirect effects on plankton. Water temperature variations regulate vertical water transport and induce mixing during cold events or stratification during heat events, even in shallow costal lagoons^3,45^. As an example, *Nitzschia* sp., often classified as a benthic Bacillariophyceae^46^, was negatively correlated with water temperature during the 2015 bloom (Fig. 4B), suggesting that colder temperatures affecting the mixing of the water column might cause resuspension of *Nitzschia* sp., increasing its abundance. On the other hand, higher water temperatures could have strengthened the stratification by modifying the physical and chemical conditions of the water column (e.g., oxygen or nutrient depletion or changes in salinity or the daily light dose) and have triggered shifts in plankton assemblages^32,34^.

Water temperature conditions were very different between 2015 and 2016. Those in 2015 were typical, with a significant winter cooling (4 °C) and a rapid temperature increase in spring (Supplementary Figure 2). On the other hand, the winter of 2016 was the warmest ever recorded in south of France, with high water temperature and no significant cooling. These conditions induced drastic changes in the structure, composition and succession of the plankton community during 2016. First, small-size ESD groups dominated (e.g. phytoplankton < 6 µm, Bacteria, heterotrophic nanoflagellates, aloricate ciliates < 20 µm and 20–27 µm) at the expanse of large-size ESD groups, that had higher abundances in 2015 (Supplementary Figure 5). Pico and nanophytoeukaryotes such as *Lohmaniella* sp. and *Strombidium* spp. (4 species) prevailed in 2016 over *Chaetoceros* sp1 and sp2, *Pseudo-nitzschia* sp. *Tiarana fusus*, *Balanion* sp., *Urotrichia* sp. that dominated the respective communities in 2015. Furthermore, water temperature exhibited more correlations with plankton taxa (Table 1; Fig. 4) in 2016 (26) than in 2015 (19), even when taking into account only the taxa found during both years. Warming promotes the dominance of small-size phytoplankton and bacteria, probably due to a volume/ratio advantage at higher temperature^37,47^, and favours the metabolism of heterotrophic organisms^30^. Thus, the warmer temperatures recorded during 2016 may have sustained the prevalence of small phytoplankton, along with small-size protist grazers such as heterotrophic nanoflagellates and small ciliates, which persisted during the winter. Under prospected global warming, the importance of small plankton organisms in shallow coastal waters is expected to increase thus leading to boost the relevance of the microbial food web and resulting in a less efficient transfer of energy to higher trophic levels.

Light was correlated significantly with the nMDS axis (Fig. 2), thus representing (after water temperature) one of the most important forcing factor of the structure of the entire plankton community. In Thau Lagoon, light was suggested as non-limiting for phytoplankton bloom initiation^19^. The present study suggested that light does not influence phytoplankton abundance (Fig. 4, low number of Spearman’s correlations between phytoplankton and light) but instead plays an important role in the composition and succession of the phytoplankton community (Fig. 3, high number of Mantel’s correlations between phytoplankton and light). Small phytoplankton cells are more efficient at utilising low light intensity; due to the smaller packaging effect, they are less penalised by self-shading^48,49^ than larger phytoplankton. Consistent with these observations, smaller cells, such as those of Cryptophyceae, Chrysophyceae and Chlorophyceae (always < 12 µm, Supplementary Figure 5), peaked during winter and early spring (Fig. 1 and Supplementary Figure 3), when light intensity was lower (Supplementary Figure 2). However, Cryptophyceae (e.g., *Plagioselmis prolonga*) are often mixotrophic and their high abundance during the winter might be due to a shift towards the heterotrophic mode. Such shift might have been favoured by the low light intensity in winter and might have resulted in Cryptophyceae consuming bacteria^50^. Larger cells, such as those of Bacillariophyceae and Dinophyceae (> 12 µm, Supplementary Figure 5), increased instead during late spring (Fig. 1 and Supplementary Figure 3). Phytoplankton succession can also be due to photoacclimation^51^. The daily dose of incident light is generally high, and from winter to spring, it does not represent a limiting factor in shallow Mediterranean coastal sites^19^. Consequently, phytoplankton succession may be influenced by the capacity to acclimate to different light conditions through the production of photosynthetic or photoprotective accessory pigments rather than being limited by light availability.

The tight connection between light and water temperature may explain the relevance of light in correlations with heterotrophic taxa. The fact that water temperature, PAR and UVBR shared links with many nodes in common supports this hypothesis (Figs. 3 and 4). During the bloom of 2015, UVBR exhibited positive correlations with planktonic taxa, and more than half of these taxa were correlated with water temperature. Air anticyclones generally increase light and air temperature^52^, consequently raising water temperature and affecting the plankton community. However, this cannot be the only explanation, as variables describing light conditions were sometimes far from water temperature in the correlation network and did not share any links with common taxa with this factor. UVBR affected the composition and taxon abundance dynamics of the plankton community in non-univocal way. The relationships with taxa varied in sign and changed according to species sensitivity and period. The positive correlations between prey taxa (lower trophic level) and UVBR may reflect harmful effects on predators. Such negative impacts of UVBR reduce predation pressure and result in positive effects on prey, as demonstrated previously^53,54^. In contrast, UVBR could indirectly trigger positive effects on phytoplankton due to photochemically induced breakdown of dissolved organic matter, which releases nutrients and enhances phytoplankton growth^55^.

The non-bloom periods during winter and early spring were characterised by high nutrient concentrations (Fig. 2). During both years, the concentrations of PO_4_^3−^, NO_3_^−^, NO_2_^−^ and SiO_2_ correlated well with various clusters (Fig. 3) and taxon abundances (Table 1; Fig. 4). A previous study suggested that Thau Lagoon is a nitrogen- and phosphorous-limited system^56^. Our results show that small opportunist phytoplankton taxa such as phytoplankton < 6 µm, Cryptophyceae and Chrysophyceae (ESD < 12 µm, Supplementary Figure 5) prevail during the non-bloom period in winter, when nutrient concentrations are high but water temperature and the daily light dose are low (Figs. 1 and 2, Supplementary Figure 3). Low nutrient concentrations and high temperature and daily doses of light are instead associated with larger phytoplankton taxa (ESD > 12 µm, Supplementary Figures 3 and 5), such as Bacillariophyceae and auto/mixotrophic Dinophyceae (Figs. 1 and 2). Usually, smaller phytoplankton cells dominate in low nutrient conditions (e.g. gyres) due to an advantage in their surface/volume ratio while larger cells prevail when the availability of nutrients is high (e.g. upwelling)^49,57,58^. In the Thau Lagoon, the level of nutrients (phosphate and nitrogen) declined during the last two decades (i.e. oligotrophication) and caused a shift in the community composition toward the dominance of small phytoplankton at the expanse of larger size taxa (e.g. Bacillariophyceae)^59^. Our results suggest that water temperature and light are more relevant than nutrients in driving seasonal changes in phytoplankton community structure, composition and succession. The role of the nutrients becomes instead more relevant for driving modifications in the decadal phytoplankton community structure.

During 2016, viruses, bacteria, heterotrophic nanoflagellates and Cyclotrichia (aloricate ciliates 20–27 µm) were connected to NO_2_^−^ (Fig. 3B,D). This result suggested enhanced activity of the microbial loop, as either the excretion of heterotrophic flagellates and Cyclotrichia or organic matter release due to viral lysis might have triggered bacterial nitrification. This process consists of bacteria using NH_4_ to produce NO_2_^−^ and then NO_3_^−^, with rapid assimilation of the last compound by phytoplankton^60^. 2016 was an unusually warm year^19^, and water temperature could also have accelerated nutrient remineralisation. The present results are in accordance with those of a previous study suggesting that the warm conditions of 2016 in Thau Lagoon favoured the co-occurrence of smaller taxa, including heterotrophic nanoflagellates, viruses and bacteria^22^.

Wind and turbidity were positively correlated (Supplementary Figure 6) and were among the six environmental nodes most connected to plankton taxa (Fig. 4A,C). During the non-bloom periods, wind (direction in 2015 and speed in 2016) and turbidity were closely related to nutrient concentrations (Fig. 2). The nMDS results illustrated consistency between wind, turbidity and nutrients. They suggested that the wind could have been responsible for the constant sediment resuspension and inputs of nutrients from the sediment to the water column from winter to spring rather than in specific and short events. In the Thau Lagoon, wind occurs frequently and can last for several weeks. It influences the ecosystem by increasing turbidity and contributing to inputs of nutrients through resuspension^16,17^. These processes were also described for other coastal ecosystems^18,61^. The supply of nutrients from sediment resuspension is fairly constant and sufficient to ensure phytoplankton growth in Thau Lagoon^16,19^. Here, sediment (and potentially nutrients) resuspension through wind seems to be more important during non-bloom periods in winter. However, weekly correlation analysis did not reveal any links between wind and nutrient concentrations (Supplementary Figure 6)^19^. The absence of significant relationships between wind and nutrients may be due to the weekly samplings, which may be inadequate for studying nutrient dynamics in systems characterised by regular resuspension and phytoplankton uptake^19^. Moreover, multiple and simultaneous mechanisms, such as precipitation and discharge from rivers, may interact to change the concentration of nutrients in the lagoon. On the other hand, wind can influence the plankton community through resuspension of benthic organisms in the water column^62,63^. The benthic Bacillariophyceae *Nitzschia* sp.^46^ and the benthic aloricate ciliate *Uronema* sp.^64^ were positively correlated with wind speed in the non-bloom period of 2016, suggesting that their abundance increased because of wind resuspension.

Occasional links connecting depth, salinity and precipitations to community composition (Figs. 2 and 3; Table 1) and taxa abundance (Fig. 4; Table 2) were observed. Depth and salinity displayed mutual positive correlations, and were negatively correlated to precipitation (Supplementary Figure 6), without displaying any particular associations with specific periods. In Thau Lagoon, the positive correlations with salinity and depth depend on seawater input through the main channel connecting the lagoon to the Mediterranean Sea. An increase in depth is generally associated with an increase in salinity due to marine water being pushed into the lagoon by southern winds. This mechanism could have two distinct effects on the plankton community composition. First, seawater input could have brought offshore taxa into the lagoon, directly modifying the food web. This phenomenon is common in marine lagoons or coastal waters subject to tides, and the composition of the plankton community depends on the balance between imports and exports^8,65^. However, in coastal zones with low tides, such as Thau Lagoon, tidal water transport is limited and often masked by other forcing factors (i.e., wind, sea currents, river inputs, or topographical constraints such as channels and natural or artificial dykes)^66,67^. The transport of plankton into the lagoon is due to currents or wind pushing marine water from the Mediterranean Sea rather than tidal action. Such factors increase depth (water level) and salinity in the lagoon. Second, salinity increases due to marine water transport could have influenced the plankton community through physiological effects. On the other hand, precipitations also change the levels of salinity in the Thau Lagoon (Supplementary Figure 6). Freshwater inputs caused by the rains reduce in fact the salinity (e.g. winter 2015) while droughts, dominated by evaporation, increase the salinity (e.g. winter 2016). Salinity exposes sensitive organisms to osmotic stress and promotes the replacement of salinity-sensitive species by salinity-tolerant taxa^68^. *Prorocentrum* sp. is known to be a salinity-tolerant genus^68,69^ and was found to be positively correlated with salinity during the bloom period of 2016 (Fig. 4D). In estuaries and coastal ecosystems subject to constant changes, salinity is an important factor influencing plankton community structure^65,70^. In terms of salinity, Thau Lagoon is relatively stable for a coastal site, and important variations are limited to strong rains, evaporation or water inputs^9,19^. The mean water residence time at the study site is approximately 50 days^71^, and the effect of salinity is therefore limited to occasional events.

The present paper highlighted that water temperature exerts stronger impacts than other environmental factors on the plankton community in a shallow coastal zone. This factor governed the composition, succession and structure of diverse plankton groups, species and trophic levels, suggesting its ubiquitous role in food web control. In shallow systems controlled by water temperature, global warming could deeply modify plankton communities. Warming is in fact expected to promote small planktonic organisms such as bacteria, picophytoeukaryotes, heterotrophic nanoflagellates and small ciliates, increasing the relevance of the microbial food web, reducing energy transfer to the higher trophic levels, and thus affecting the whole ecosystem productivity.

## Methods

### Study site and monitoring design

Monitoring took place in Thau Lagoon (Supplementary Figure 1), a productive marine lagoon located on the French coast of the northwestern Mediterranean Sea (43°24′00″ N, 3°36′00″ E). It is a 75 km^2^ shallow lagoon with a mean depth of 4 m and a maximum depth of 10 m (excluding a 32-m-deep depression) and is connected to the Mediterranean Sea by two main channels. Thau Lagoon is a mesotrophic, phosphorus- and nitrogen-limited system^56^ with a turnover rate of 2% (50 days). Salinity ranges from 34 to 38 PSU, and high-amplitude temperature fluctuations occur throughout the year, ranging from 4 °C in the winter to 30 °C in the summer^19,29^. The lagoon is frequently exposed to high wind speeds throughout the year^17,19^. Thau Lagoon hosts economically important activities, mainly oyster farms representing 10% of French production.

The water column was monitored at the fixed station of the Coastal Mediterranean Thau Lagoon Observatory (43°24′53″ N, 3°41′16″ E)^72^ at the Mediterranean platform for Marine Ecosystem Experimental Research (MEDIMEER) in Sète. The fixed monitoring station is located less than 50 m from the main channel connecting the lagoon to the Mediterranean Sea, where the water residence time is short (20 days)^71^. The water depth at the monitoring station was 2.5–3 m, and samplings took place at a depth of 1 m. The monitoring lasted from winter to spring for two consecutive years, i.e., from January 8 to May 12, 2015, and from January 12 to June 14, 2016. The monitoring consisted of (1) high-frequency (every 15 min) recordings of meteorological, hydrological, and chlorophyll *a* (Chl *a*) fluorescence data using automated sensors and (2) weekly sampling using Niskin bottles or plankton nets (20 µm) to quantify nutrient concentrations, plankton diversity, and plankton abundances. Weekly sampling for nutrients and all organisms was carried out in the morning between 09 and 10 a.m. Some of the data used in the present study were already published in previous manuscripts^19,22^. Thus, details on acquisition methods can also be found in these manuscripts, as specified below.

Hydrological and meteorological data, including air and water temperature, wind speed and direction, PAR (400–700 nm), UVBR (280–320 nm), salinity and turbidity, as well as Chl *a* fluorescence and nutrient concentrations, including those of nitrite (NO_2_^−^), nitrate (NO_3_^−^), phosphate (PO_4_^3−^) and silicon dioxide (SiO_2_), are available in Trombetta et al. (2019)^19^. Phytoplankton abundance and diversity data are available in Trombetta et al. (2019, 2020)^19,22^. Virioplankton, bacterial, heterotrophic nanoflagellate, aloricate ciliate, and tintinnid abundances and diversity data are available in Trombetta et al. (2020)^22^. The atmospheric pressure, humidity, precipitation, depth, large heterotrophic Dinophyceae, rotifer, mesozooplankton larva, and copepod data presented in this study were not published elsewhere.

### Hydrological, meteorological, nutrient, and chlorophyll *a* measurements

Hydrological and meteorological data were recorded every 15 min (except precipitation). Depth was recorded every 15 min with an STPS sensor (NKE Instrumentation). Atmospheric pressure and humidity were recorded using a Professional Weather Station (METPAK PRO, Gill Instruments). Daily precipitation was retrieved from the Météo-France open access database^73^, referring to a meteorological station located 2 km away from the monitoring station. PAR Daily Light Integral (DLI) was calculated following Eq. (1) where Σ PAR is the sum of PAR measured during the day (96 measures) and ∆t is the time interval between two measurements (900 s).

1 $$ {\text{DLI}} = \sum PAR \times \Delta t $$

Supplementary Figure 2 shows the daily mean dynamics of the environmental parameters and PAR DLI.

To investigate the effects of environmental parameters on weekly community composition, the mean of all environmental variables between 09 and 10 a.m. was calculated for days corresponding to the sampling dates.

Nutrient concentrations, i.e., those of PO_4_^3−^, NO_2_^−^, NO_3_^−^ and SiO_2_, were measured using an automated colorimeter (Seal Analytical) as described in Trombetta et al. (2019)^19^. Chl *a* fluorescence was measured at high frequency (15 min) using a WETLAB sensor, and the data and methodological details are available in Trombetta et al. (2019)^19^.

### Plankton identification and abundance

The diversity and abundances of (1) viruses; (2) non-pigmented planktonic cells < 1 µm, including archaea, heterotrophic bacteria, and chemosynthetic bacteria (hereafter called “bacteria”); (3) phytoplankton; (4) HFs; (5) aloricate ciliates and tintinnids; (6) large heterotrophic Dinophyceae; and (7) metazooplankton were determined. For each of these groups (except large heterotrophic Dinophyceae and metazooplankton), the methods of abundance estimation were described in Trombetta et al. (2020)^22^. Large heterotrophic Dinophyceae and metazooplankton were sampled using a plankton net (20 µm) towed at the sampling site over 424 L and then fixed with stabilised formaldehyde (4% final concentration) in 110 mL glass bottles and stored in the dark at 4 °C until analysis. Large zooplankton sampling took place in 2016 but not in 2015. Large heterotrophic Dinophyceae and metazooplankton taxon abundances were estimated using a binocular loop. Replicates of 2–5 mL were taken and placed into a Bogorov counting chamber. Zooplankton individuals were identified and counted under a stereo microscope (Olympus SZX7). Autotrophy, mixotrophy and heterotrophy of taxa was determined using previous evidence from the literature^74,75^.

Supplementary Table 1 summarises all data used in this manuscript, with the methods used and if already published elsewhere.

### Clustering of plankton

The abundance of planktonic groups/taxa/species was clustered based on (1) ESD and (2) taxonomy. Clustering enabled statistical analysis of specific groups of interest and comparisons of their differential responses to the various environmental parameters.

First, the abundance of planktonic groups/taxa/species was expressed according to clusters based on ESD. Such a clustering method was applied in a previous study, and the clusters identified and used below can be found in Trombetta et al. (2020)^22^.

Second, new clusters were made using group/taxon/species taxonomic membership. Bacteria with high nucleic acid (HNA) and low nucleic acid (LNA) were pooled into the ‘bacteria’ cluster. The different size classes of HFs were pooled into the ‘HF’ cluster. The two heterotrophic Dinophyceae species identified were pooled into the ‘heterotrophic Dinophyceae' cluster. Phytoplankton taxa were pooled into seven clusters according to taxonomy. The clusters were ‘Bacillariophyceae', ‘auto/mixotrophic Dinophyceae', ‘Cryptophyceae’, ‘Chrysophyceae’, ‘Chlorophyceae’, and ‘other phytoplankton’. This last phytoplankton cluster included the few taxa left, which belonged to different groups with only one or two representatives (i.e., Prymnesiophyceae, Prasinophyceae, and Euglenophyceae). Smaller phytoplankton groups identified with flow cytometry were pooled into a ‘phytoplankton < 6 µm’ cluster. Aloricate ciliates were pooled into five clusters according to order. The clusters were ‘Choreotrichida’, ‘Oligotrichida’, ‘Prorodontida’, ‘Cyclotrichiida’, and ‘other aloricate ciliates’. This last aloricate ciliate cluster included the few taxa left, which belonged to different groups with only one or two representatives (i.e., Oligohymenophorea, Ceramiales, unidentified spirotrich, and unidentified aloricate ciliates). Loricate ciliates (tintinnids) were pooled into six clusters according to family. The clusters were ‘Codonellidae’, ‘Metacyclididae’, ‘Tintinidae’, ‘Ptychocylididae’, ‘Codonellopsidae’ and ‘other tintinnids’. This last tintinnid cluster included the few taxa left, which belonged to different groups with only one or two representatives (i.e., Ascampbelliellidae and unidentified tintinnids). Metazooplankton were divided into three groups: ‘mesozooplankton larvae’, ‘rotifers’, and ‘copepods’.

### Statistical analysis and network visualisation

The bloom and non-bloom periods in 2015 and 2016 were previously identified in Trombetta et al. (2019)^19^ based on daily mean Chl *a* fluorescence, a measure of phytoplankton biomass. The daily net growth rate was calculated as the difference in phytoplankton biomass between two consecutive days, with negative values indicating biomass loss whereas positive values stand for biomass increase. A bloom occurs when there are at least two consecutive days with positive growth rates and the sum of the net growth rates over at least five consecutive days is positive; The non-bloom starts the day before five consecutive days with negative growth rate^19^. The authors found that different environmental variables were involved in Chl *a* dynamic depending on the period (bloom or non-bloom). Furthermore, Trombetta et al. (2020)^22^ considered the same periods and found different food web network structures depending on the period (bloom or non-bloom). Consequently, the same bloom and non-bloom dataset separation was used in the present study to investigate the environmental forcing factor associated with changes in plankton community composition and structure during these productive periods.

nMDS ordination was applied to classify planktonic taxa based on temporal and environmental similarities. Community composition from winter to spring, along the axis of dates, and during productive periods was investigated for the years 2015 and 2016. To determine which environmental factors drive plankton community assembly, the environmental variables were ordinated, and their correlation scores with nMDS axes were calculated using the *envfit* function in R (*vegan* package, version 2.4-2).

Mantel’s test was used to identify the significant correlations between environmental factors and plankton community composition according to each ESD and taxonomic cluster. This study aimed to identify the potential role of environmental variables in driving plankton community composition during the bloom and non-bloom periods in both years. Correlations were calculated between two matrices: (1) one of environmental parameters and (2) one of abundances for the ESD or taxonomic clusters. Mantel’s tests were performed on each environmental parameter and ESD/taxonomic cluster pair. Only environmental factors significantly correlated with the nMDS axes were used in Mantel’s test.

Spearman’s rank correlation was applied to identify significant mutual changes between environmental factors and the abundance of taxa in the bloom and non-bloom periods in both years. Correlation tests were carried out for each environmental parameter and taxon abundance pair. A Monte Carlo resampling procedure was applied, and 9999 iterations were used to quantify the *p*-values.

Outcomes of Mantel’s and Spearman’s tests were visualised through networks to clarify the emergence of patterns at the scale of the entire plankton community. A network is a spatial representation of associations, marked by lines (called ‘edges’) linking two entities (called ‘nodes’). The nodes are the environmental factors, taxa, and ESD or taxonomic clusters, and the edges represent significant correlations (*p* < 0.05).

## Supplementary Information

Supplementary Information.
