# Supplementary material for: Co-occurrence networks reveal the central role of temperature in structuring the plankton community of the Thau Lagoon
Source: Sci Rep. 2021 Sep 3;11:17675. doi: 10.1038/s41598-021-97173-y (PMC8417261; doi:10.1038/s41598-021-97173-y)
Supplement: Supplementary file 1 — Supplementary Information. [file 41598_2021_97173_MOESM1_ESM.docx]

**SUPPLEMENTARY MATERIAL**


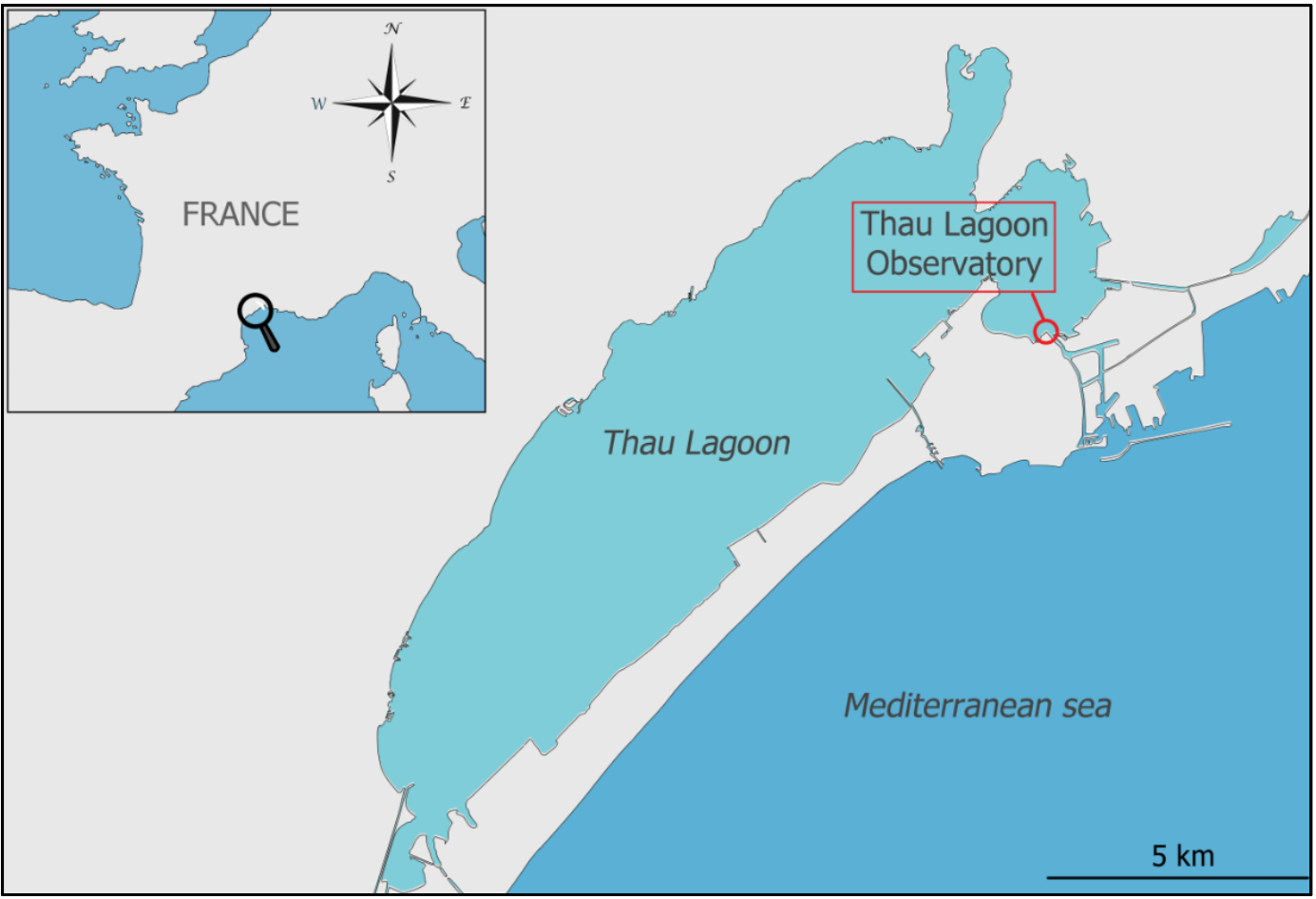


**Supplementary Figure 1:** Thau Lagoon and the sampling station. Grey indicates land, and blue indicates water bodies. Figure was drawn by Justine Courboulès.


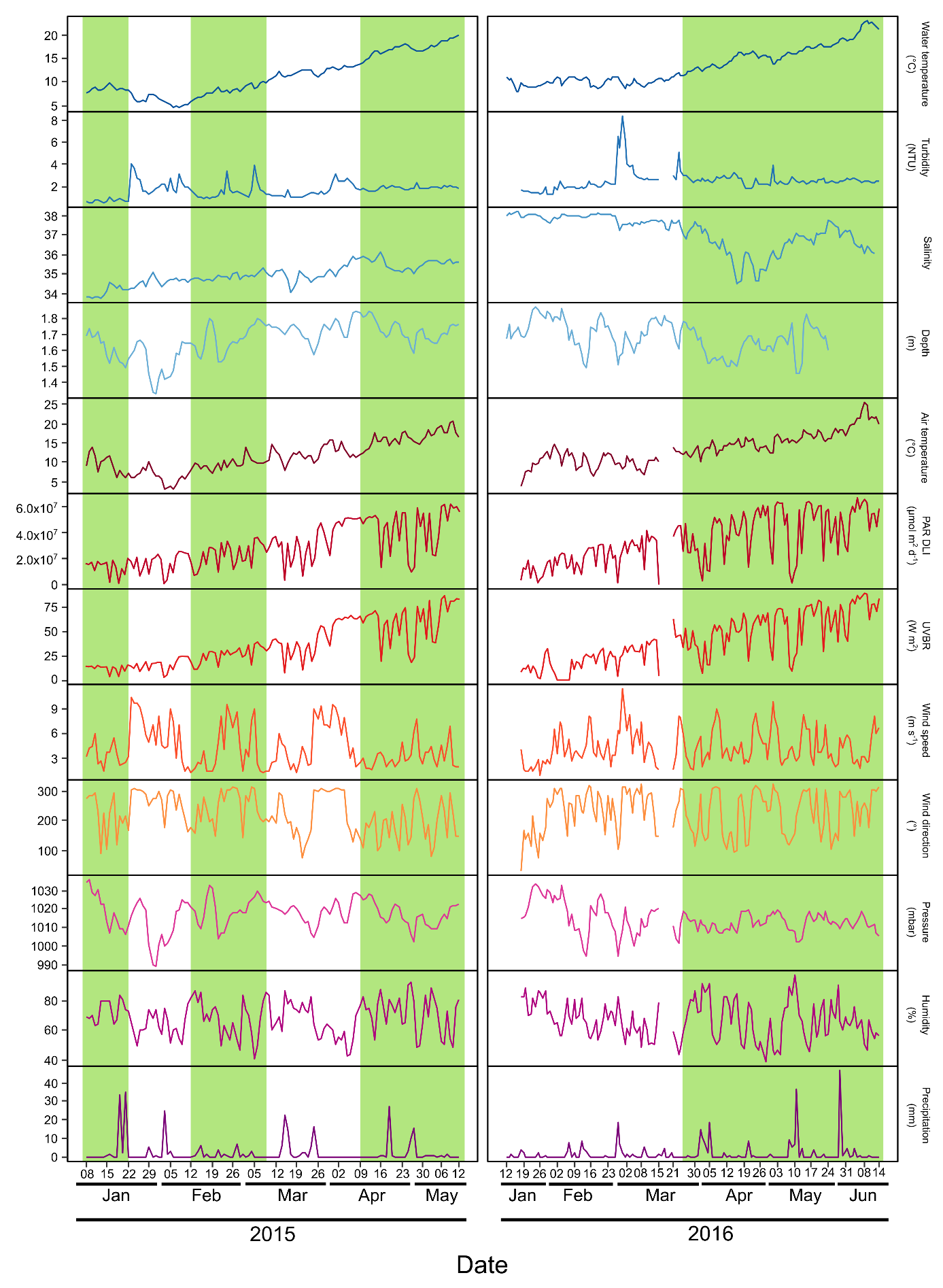


**Supplementary Figure 2:** Daily dynamics of environmental parameters in 2015 (left) and 2016 (right). The green background represents bloom periods, and the white background represents non-bloom periods.

**
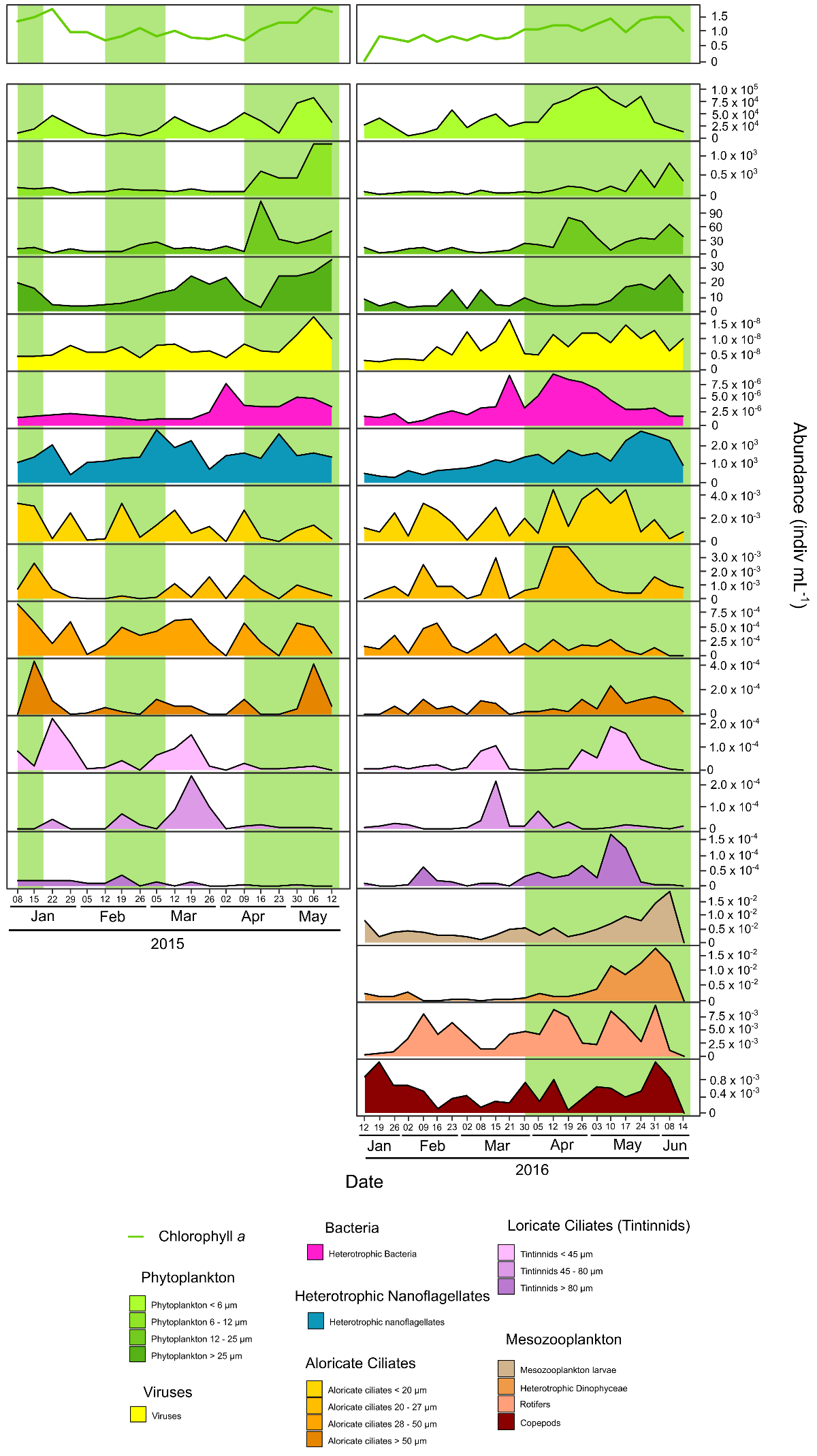
**

**Supplementary Figure 3:** Dynamics of Chl *a* concentration and planktonic ESD group abundance in 2015 (left) and 2016 (right). Green and white backgrounds represent bloom and non-bloom periods, respectively.


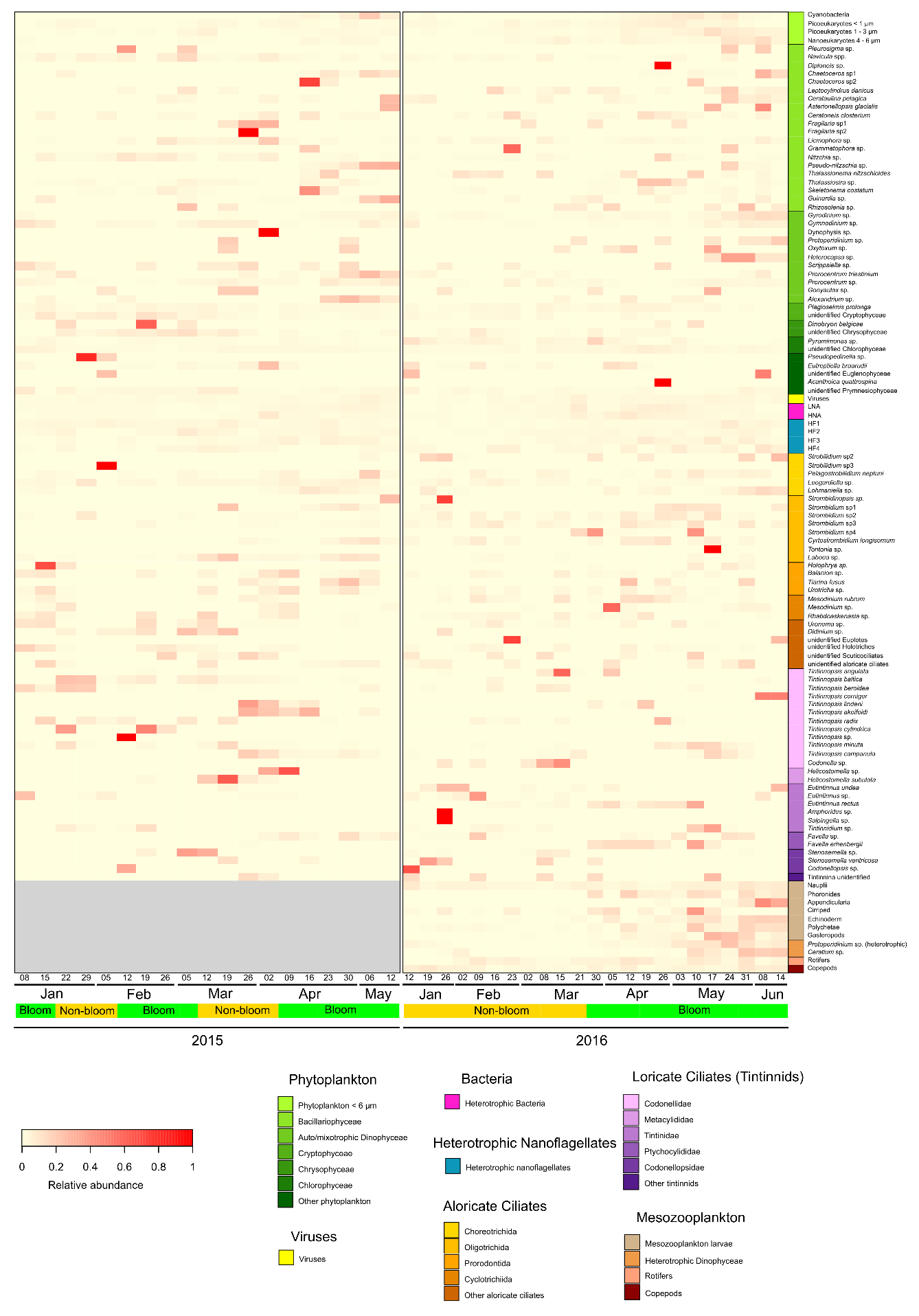


**Supplementary Figure 4:** Temporal relative abundances of taxa grouped by taxonomy in 2015 (left) and 2016 (right). Relative abundances were calculated in both years for each taxon on each date.

**
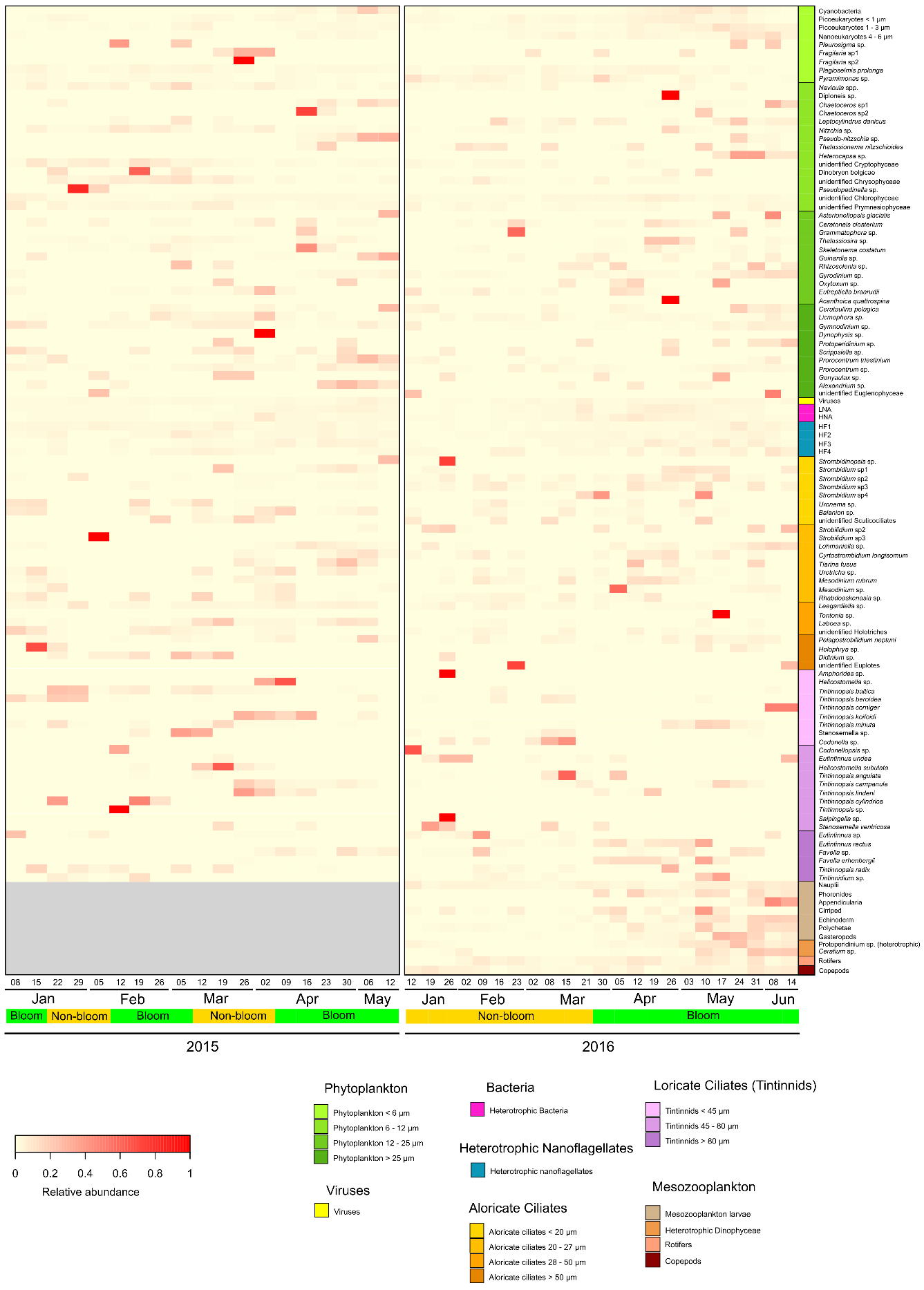
**

**Supplementary Figure 5:** Temporal relative abundances of taxa grouped by ESD in 2015 (left) and 2016 (right). Relative abundances were calculated in both years for each taxon on each date.

**
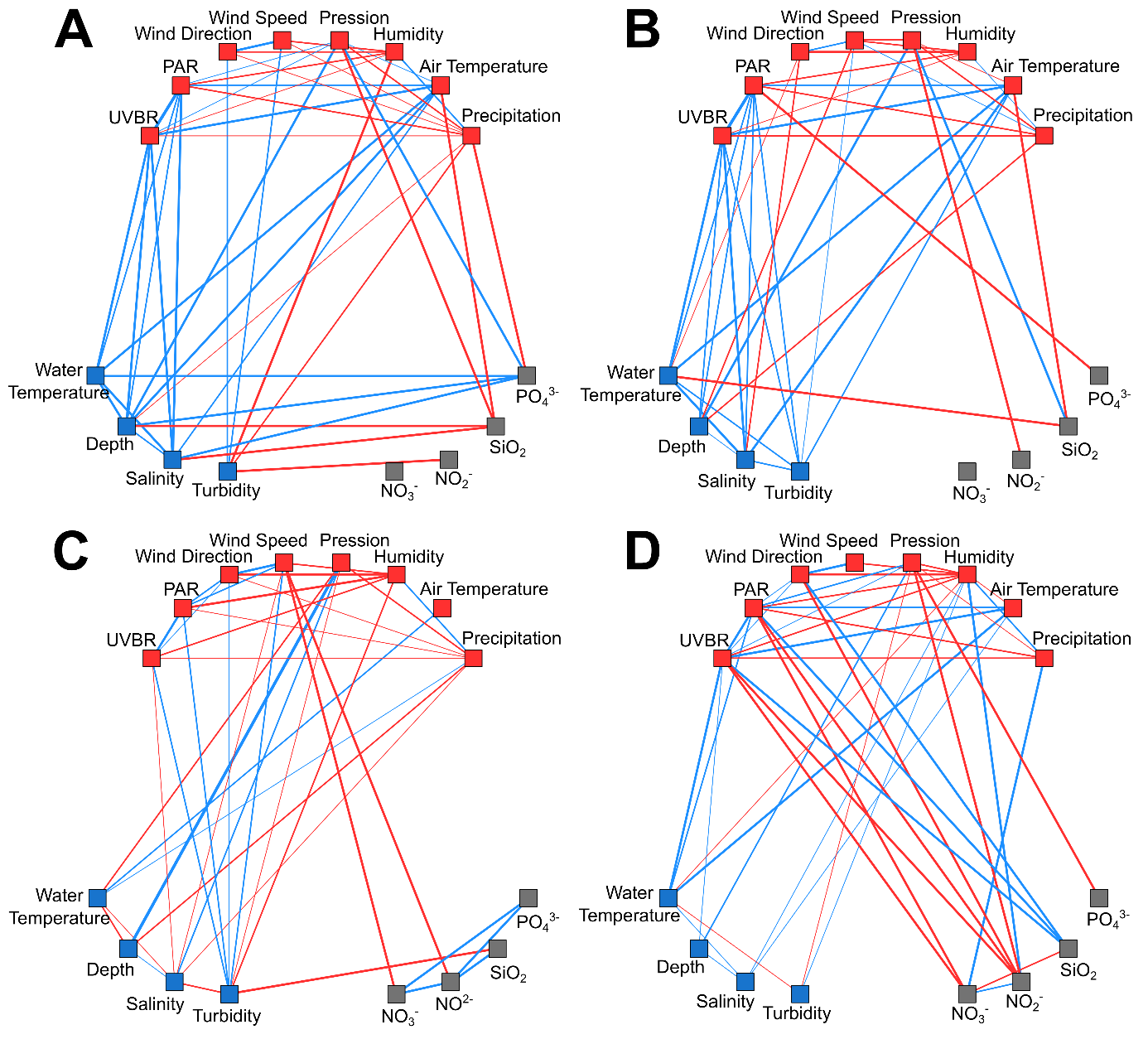
**

**Supplementary Figure 6:** Co-occurrence networks constructed using Spearman’s correlations between environmental parameters and nutrient concentrations. Red nodes are meteorological parameters. Blue nodes are hydrological parameters. Grey nodes are nutrient concentrations. Blue and red edges represent positive and negative correlations, respectively.

**Supplementary Table 1: Summary of the data presented in the study, their acquisition frequency, used methods and references for already published datasets.**

| Category of data | Type of data | Type of instrument | References |
| --- | --- | --- | --- |
| Hydrological | Depth | Sensor: NKE STPS | Present study |
|  | Salinity |  | ^19^ |
|  | Water temperature |  | ^19^ |
|  | Turbidity | Sensor: ECO FLNTU fluorometer (Wetlabs) | ^19^ |
| Meteorological | Precipitation | Météo-France open access database  https://donneespubliques.meteofrance.fr/ | Present study |
|  | Pressure | Sensor: Professional Weather Station (METAPAK PRO, Gill Instruments) | Present study |
|  | Humidity |  | Present study |
|  | Air temperature |  | ^19^ |
|  | Wind speed |  | ^19^ |
|  | Wind direction |  | ^19^ |
|  | PAR (400-700 nm) | Light sensor: Skye Instruments | ^19^ |
|  | UVBR (280-400 nm) |  | ^19^ |
| Biological | Chl *a* fluorescence | Sensor: ECO FLNTU fluorometer (Wetlabs) | ^19^ |
| Nutrients | Nutrient concentrations (NO_2_^-^, NO_3_^-^, PO_4_^3-^, SiO_2_) | Sampled with a Niskin bottle and analysed using an automated colorimeter (Seal Analytical) | ^19^ |
| Plankton abundance | Large heterotrophic Dinophyceae (2 taxa) | Sampled with a plankton net and analysed using a binocular loop | Present study |
|  | Mesozooplankton (9 groups/taxa) |  | Present study |
|  | Virioplankton (1 group) | Sampled with a Niskin bottle and analysed using an epifluorescence microscope (Olympus AX-70) | ^22^ |
|  | Heterotrophic nanoflagellates (4 groups) |  | ^22^ |
|  | Bacteria (2 groups) | Sampled with a Niskin bottle and analysed using a flow cytometer (FACSCalibur, Becton Dickinson) | ^22^ |
|  | Phytoplankton < 6 µm (4 groups) | Sampled with a Niskin bottle and analysed using optical microscopy (Olympus IX-70) | ^19,22^ |
|  | Phytoplankton > 6 µm (42 groups/taxa) |  | ^19,22^ |
|  | Ciliates (57 groups/taxa) |  | ^22^ |
